# Japanese Herbal Medicine (Kampo) as a Possible Treatment for Ischemia With Non-obstructive Coronary Artery Disease

**DOI:** 10.7759/cureus.38239

**Published:** 2023-04-28

**Authors:** Hiroki Teragawa, Chikage Oshita, Yuko Uchimura

**Affiliations:** 1 Department of Cardiovascular Medicine, JR Hiroshima Hospital, Hiroshima, JPN

**Keywords:** microvascular angina, vasospastic angina, kampo, japanese herbal medicine, coronary microvascular dysfunction

## Abstract

Patients presenting with the syndrome of symptoms and signs suggesting ischemic heart disease but found to have no obstructed coronary arteries (INOCA) are increasingly recognized. Although there are non-invasive tests for the diagnosis of INOCA, such as transthoracic Doppler echocardiography, positron emission tomography, and cardiac magnetic resonance imaging to evaluate increased blood flow with adenosine and other agents, the diagnosis of INOCA by coronary angiography with the coronary spasm provocation test and coronary microvascular function evaluation using pressure wires has become the gold standard, but it is not well established in the treatment of INOCA. Despite the lack of objection to lifestyle modification and the use of coronary dilators, mainly calcium-channel blockers, for conditions involving epicardial coronary artery spasm, there is no entirely effective long-term treatment for microvascular spasm or coronary microvascular dysfunction. Although some combinations of drugs have been empirically administered in certain cases, it is difficult to conclude that they are sufficiently effective. Recently, it has been reported that some Japanese herbal medicines (Kampo) have been effective in the treatment of INOCA. In order to increase the knowledge on the treatment of INOCA, this review focuses on the effects of Japanese herbal medicine on INOCA and its presumed mechanisms and problems.

## Introduction and background

Introduction of diagnosis and treatment of ischemia with non-obstructive coronary artery disease (INOCA)

INOCA has been widely recognized and has become an important part of ischemic heart disease because patients with INOCA do not always have a good prognosis [1,2]. There have been several non-invasive examinations for investigating the presence of coronary microvascular dysfunction (CMD), such as transthoracic Doppler echocardiography, positron emission tomography, and cardiac magnetic resonance imaging [3]. With the combination of these examinations and coronary computed tomographic angiography to exclude the presence of organic coronary stenosis, the diagnosis of INOCA may be possible [3]. However, a certain percentage of vasospastic angina (VSA), which is characterized by transient epicardial coronary vasoconstriction on coronary angiography (CAG), combined with chest symptoms and/or ST-T changes of electrocardiogram [4], must be included in INOCA [3,5]. Furthermore, the CorMicA study [6] demonstrated that CAG plus coronary functional test (CFT) and treatment based on the results of these examinations significantly improved chest pain status at six months, compared with conventional coronary angiography in patients with INOCA. Considering these findings, CAG and CFT, including the spasm provocation test (SPT) to assess the presence of VSA and/or microvascular spasm (MVS) and coronary microvascular function (CMVF) to assess the presence of CMD, have become the mainstream method for the evaluation of INOCA [3,7], despite the uncertainty of whether SPT or CMVF should be performed first at this stage. The European consensus document of INOCA [3] recommended performing CMVF first, followed by SPT for the evaluation of VSA. This method may reduce the positivity for SPT because the coronary arteries are evaluated in CMVF after they are maximally dilated by nitroglycerin (NTG). On the contrary, at least severe VSA may not be missed because of the short duration of NTG and severe VSA can be induced even after NTG administration [8,9]. The frequency of VSA, MVS, and CMD may vary by race [10,11], and which endotype of INOCA treatment is preferred may vary by region. Future studies may clarify this issue.

In the diagnosis of INOCA, VSA was defined as >90% narrowing of the epicardial coronary arteries on angiography during SPT and the presence of characteristic chest pain and/or ST-segment deviation identified via electrocardiography (ECG) [12,13]. MVS was defined as the absence of angiographic coronary spasm accompanied by characteristic chest pain and ST-T ECG changes during SPT [3,14]. CMD was defined as the index of microcirculatory resistance of ≥25 units or coronary flow reserve of <2.0 [3,7]. Importantly, these VSA, MVS, and CMD may all overlap [1,7,15], which is thought to complicate the condition with no small effect on pathogenesis and treatment.

On the contrary, the treatment of INOCA does not appear to be well established at this time. An important possible reason for this is that the pathogenesis of INOCA is heterogeneous [3]. In general, nothing needs to be said about smoking cessation, exercise, and stress reduction as treatments for ischemic heart disease [3,7]. Specifically, smoking is one of the risk factors [16], including prognosis, in VSA [17], and smoking cessation is considered important in the treatment of VSA [12].　

In the drug treatment of VSA, it is needless to say that the use of coronary dilators, mainly calcium-channel blockers (CCB), is important, and the number of coronary dilators, such as long-acting nitrate and/or nicorandil, increased when anginal attacks occur under medications. However, intractable VSA, in which anginal attacks occur despite taking two kinds of coronary dilators, exist in certain patients, and they occurred in 13.7% of patients with VSA [12]. When taking drugs, it is necessary to consider the time of day when anginal attacks are most likely to occur and to adjust the medication and time of day, as twice a day is more effective than once a day [18]. There are reports that brand-name drugs are more effective than generic drugs [19].

MVS is considered a condition of microvascular vasoconstriction, and the main drugs are those with microvascular-dilating effects, such as CCB and nicorandil. However, nitroglycerin is considered less effective [9]. The current drug therapy for CMD is as follows: in addition to statins and renin-angiotensin system inhibitors to improve microcirculation, beta-blockers, CCBs, nicorandil, ranolazine, ivabradine, and trimetazidine are also used to treat MVS. Trimetazidine is listed in the microvascular angina section of the expert consensus document [3]. In daily clinical practice, we have often experienced that VSA alone is not so difficult to treat, but in cases of MVS or CMD, or when VSA is complicated with these conditions, patients often experience frequent chest symptoms even when taking vasodilators. Thus, it is urgent to establish an effective treatment for microvascular disorders such as MVS and CMD as therapeutic agents for INOCA.

## Review

Japanese herbal medicines (kampo) for the treatment of INOCA

Kampo medicines are composed of “crude drugs” that are parts of plants, animals, or minerals found in nature that have medicinal properties, usually in combination with several others. Thus, Kampo has a variety of pharmacological effects, such as tonifying, warming, cooling, detoxifying, and sedative effects [20,21]. Especially, regarding the sedative effect of Kampo, some kinds of Kampo medicines could cross the blood-brain barrier and exert pain-relieving and anti-depressive effects by binding central nervous system receptors in currently uninvestigated ways, whether or not similar to the mechanisms of action of some of the standard drugs used in psychiatry. For this reason, they have additional pain-relieving and anti-depressive effects [20,21].

Kampo medicine has been used as an established therapeutic agent in several areas [22-26]. However, in the field of cardiovascular medicine, some Kampo medicines have been considered possible cardiovascular treatment, especially for heart failure [20,27,28]. Even in the field of INOCA, case reports have shown the effect of some Kampo medicines on the relief of chest symptoms in patients with INOCA [29-37]. Kampo's potential as a therapeutic agent in INOCA has received much attention [38], and a recent guideline has included it as one of the therapeutic agents [39]. Herein, we presented several Kampo medicines that are effective in patients with INOCA.

Mokuboito

Mokuboito acts to dissipate and remove stagnant fluid from the body. Because of this action, it is used to treat edema and wheezing caused by heart or kidney disease. The presence of “stuffiness under the heart (Shinkahiken)” or a tight, board-like feeling in the solar plexus is a sign that the drug is being used. Because of its effectiveness, it is often used as a treatment for heart failure [20,27].

In an animal study using porcine coronary arteries subjected to acetylcholine provocation, Mokuboito, and Tokishakuyakusan are investigated for their ability to inhibit coronary spasm [40]. Although these drugs were not shown to inhibit coronary vasospasm, Mokuboito, and Tokishakuyakusan together tended to inhibit coronary spasm. In this study, every five animals were treated with Mokuboito and Tokishakuyakusan, and there was a possibility of a type 2 error.

There is a case report of a 50-year-old woman with VSA who used sublingual NTG 20 times a day despite taking a CCB and a long-acting nitrate. When she was treated with Mokuboito, she no longer needed NTG sublingually (Table *1*) [29].

**Table 1:** Case series showing some effectiveness of Kampo medicines on the relief of chest symptoms in patients with INOCA. CCB, calcium-channel blocker; F, female; INOCA, ischemia with non-obstructive coronary artery disease; KBG, keishibukuryogan; M, male; MVA, microvascular angina; No., number; NTG, nitroglycerin; Ref, reference; SGS, shigyakusan; VSA, vasospastic angina.

Kinds of Kampo	Case								Authors’ name/Ref No.
	Age	Sex	Diagnosis	Treatment before	Dose of Kampo	Treatment after	Effect	Time to onset of effect	
Mokuboito	50	F	VSA	CCB, long-acting nitrate (details unknown), sublingual NTG: 20 times/day	7.5 g/day	Probably the same as before administration	No need for sublingual NTG	details unknown	Fukushima et al. [29]
										Saibokuto	48	F	VSA	Benidipine 4 mg/day, isosorbide dinitrate 40 mg/day, sublingual NTG: 3–4/day	7.5 g/day	Same prescription as before	Less need for sublingual NTG , continuing for 2 years	2 weeks later	Shudo et al. [34]
65	M	VSA	Amlodipine 5 mg/day, isosorbide dinitrate 40 mg/day, sublingual NTG: 3–4/2 weeks	7.5 g/day	Same prescription as before	Less need for sublingual NTG, continuing for 2 years	2 weeks later	Shudo et al. [34]											
									61		F	VSA	Diltiazem R 2 capsules/day, amlodipine 5 mg/day, nicorandil 15 mg/day	7.5 g/day →5.0 g/day	Same prescription as before	Less need for sublingual NTG, continuing for 2 years	2 days later	Shudo et al. [34]
45	M	VSA	Nicorandil 15 mg/day, isosorbide dinitrate 40 mg/day, sublingual NTG	7.5 g/day	Isosorbide dinitrate 5 mg/day	Headache disappeared, no chest pain, but chest discomfort remained	18 days later	Shudo et al. [35]										
									69		M	VSA	Nicorandil 15 mg/day, isosorbide dinitrate 40 mg/day, sublingual NTG	7.5 g/day	Benidipine 8 mg/day	Headache disappeared, anginal attacks almost disappeared	36 days later	Shudo et al. [36]
isosorbide dinitrate 20 mg/day																		
Keishibukuryogan	48	F	VSA	Diltiazem (dose unknown) , isosorbide dinitrate 40 mg/day	5.0 g/day	Isosorbide dinitrate 40 mg/day	Dramatic decrease in anginal attacks: None in 4 years	Relatively rapid improvement	Naito et al. [32]	
											Keishibukuryogan + Shigyakusan	73	M	VSA	Benidipine 8 mg/day, isosorbide dinitrate 40 mg/day, nicorandil 15 mg/day	KBG 5.0 g/day, SGS 5.0 g/day	Probably the same as before administration	Complete disappearance of chest symptoms	3 months later	Yamazaki et al. [37]
58	M	VSA	Benidipine 8 mg/day, isosorbide dinitrate 40 mg/day, nicorandil 15 mg/day	KBG 2.5 g/day, SGS 2.5 g/day	Probably the same as before administration	Complete disappearance of chest symptoms	3 months later	Yamazaki et al. [37]												
									Karogaihakuhangeto			47	F	VSA	Diltiazem R 300 mg	Original blended	Western medications could be discontinued	Dramatic improvement of chest symptoms, angina-free for 8 months	3 days later	Suzuki et al. [31]
Tokito	50	F	MVA	Sublingual NTG	Dose information is not available.	Sublingual NTG	Improvement of chest symptoms	1 months later		Namiki et al. [33]										
											Daikankyoto										72	F	MVA	None	Original blended	Daisaikoto, Keishibukuryogan and amlodipine 5 mg	Chest symptoms have improved, but the diarrhea has caused the change to another drug.	Prompt	Kimura et al. [30]

Hangekobokuto

It is used for patients who are anxious or nervous, feel heavy or blocked up, feel something stuck in their throat, cannot clear a cough, have palpitations, are anxious, cannot sleep well at night, and have a weak stomach. Koboku, which is one of the ingredients of Hangekobokuto, contains magnolol, honokiol, etc., and has a comprehensive vasodilating action by inhibiting platelet aggregation, acting as a CCB and increasing cyclic guanosine monophosphate via nitric oxide [36]. In addition, stress is also considered a factor that influences the onset and exacerbation of VSA [41] and may be effective when stress is involved in attacks.

Saibokuto

Saibokuto is a fixed-dose combination of Shosaikoto and the above-mentioned Hangekobokuto, and it is said to have swelling-improving, diuretic, and anti-inflammatory effects. Saibokuto is used considering the symptoms of oppression from the anterior chest to the pharynx, which are treated by Hangekobokuto, and other abdominal symptoms, which are treated by Shosaikoto.

Three case reports, which have been published by the same author [34-36], showed that a total of five patients with VSA experienced an improvement in their symptoms when combined with the standard drugs (Table *1*). Some patients who could not take multiple coronary dilators because of side effects such as headaches had reduced the dose of coronary dilators, and their quality of life improved significantly after the introduction of this drug [34,36].

On the contrary, Saibokuto contains Shosaikoto and has some side effects such as interstitial pneumonia, pseudo-aldosteronism, and hepatic dysfunction; thus, it is necessary to reduce the dose or switch to Hangekobokuto as described above once symptoms have stabilized [34].

Keishibukurhogan

In Kampo medicine, blood that is polluted, stagnant, or clotted to the detriment of the body is called “Oketsu,” and this status is thought to be associated with microcirculatory insufficiency, hyperviscosity, hypercoagulability, and hyperplatelet aggregation. In VSA, the late-night to early-morning period, when chest pain occurs most frequently, is considered the time of day when “Oketsu” is the most severe because of rest and dehydration. Blood loss may be involved in the pathogenesis of VSA [32]. Keishibukuryogan is a representative of Kampo medicine to improve the “Oketsu” status.

Many animal experiments and clinical studies have been conducted to confirm the effect of Keishibukuryogan on blood circulation and other “Oketsu”-related dysfunction [42-47]. Keishibukuryogan has a vasodilatory effect due to the increased nitric oxide (NO) production, a protective effect on the endothelial function against oxidative stress, an inhibitory effect on platelet aggregation, and an anti-inflammatory effect [47], and these effects may be protective against vascular dysfunction, leading to the anti-ischemic effects for INOCA.

There is a case report of a 48-year-old woman with an intractable VSA, who could not receive adequate doses of coronary vasodilators because of severe headache. Her chest symptoms disappeared for four years when she took a combination of an isosorbide dinitrate patch and Keishibukurhogan [32]. In addition, two patients in whom chest symptoms were well controlled by the combination of Keishibukuryogan and Shigakusan will be reported in the next section on Shigyakusan (Table *1*) [37].

Shigyakusan

Shigyakusan is one of the Kampo medicines for irritability, nervousness, and gastrointestinal symptoms resulting from an internal build-up of emotions that are not dissipated outwardly. It improves not only chest and side distension (a feeling of resistance and tenderness in the right side of the abdomen), rectus abdominis muscle tension, and bloating, but also mental symptoms such as anxiety, insomnia, and irritability, and improves various illnesses and symptoms such as acute or chronic gastritis, gastric ulcer, cholecystitis and gallstones, neurosis, hysteria, and others.

Currently, no study has reported that Shigyakusan alone improves the symptoms of INOCA. Stress may cause the worsening symptoms of VSA [41], and there is a case report of two patients with VSA, whose chest symptoms have been effectively controlled by the combination of Shigyakusan and Keishibukuryogan, which improves “Oketsu” symptoms as described above (Table 1) [37].

Karogaihakuhangeto

The target patients are those who complain of pain in the heart, sternum, or orbit, radiating pain in the back, bronchial asthma, cough, sputum, dyspnea, chest pain, and vomiting. Currently, no medical extract formulation is available, and it has the disadvantage of being difficult to swallow. It is said to be good for patients with ischemic heart disease who have chest pain when breathing cold air or when chest pain occurs after a sudden movement.

Basic research has shown that karogaihakuhangeto inhibits platelet aggregation [31], and since platelet aggregation is involved in the aggravation of VSA [48], karogaihangeto may be effective in improving VSA symptoms. A case report presented a patient with VSA who completely discontinued the standard medications after taking this herbal remedy (Table 1) [31].

Tokito

Tokito is used for a relatively weak person with poor circulation and physical strength, who is feeling cold and has pain in the chest, abdomen, and back. Thus, it has been used for the treatment of various diseases and thoracic and abdominal issues, such as cardiac neuropathy, intercostal neuralgia, and abdominal diseases such as peptic ulcer and chronic gastritis. It also relieves stress and may be effective for INOCA, especially MVS, and CMD, since stress is involved in the symptoms of INOCA [3,41]. A case report presented a case of microvascular angina in which the frequency of attacks decreased after taking Tokito (Table 1) [33].

Daikankyoto

Daikankyoto is sometimes prescribed for angina pectoris or other conditions suggestive of myocardial infarction, as well as for impulsive pain in the legs, but since there is no medical extract formulation of this herb, it is prepared by individual herbalists.

It has been reported to be effective for microvascular angina [30], but the drug led to diarrhea as an immediate side effect, and the prescription was changed to Daisaikoto, Keishibukuryogan, and amlodipine 5 mg. The efficacy of the drug could not be evaluated (Table *1*).

Present status and future challenges of kampo as a potential drug for INOCA

As mentioned above, Kampo medicines may improve the symptoms of patients with INOCA by improving stress and “Oketsu” status. At present, despite case reports of patients who have discontinued the standard medications for INOCA [31], this is an adjunctive treatment to the standard medications that have been taken in accordance with standard guidelines [7,12,39]. In cases of severe side effects, such as headache, with conventional coronary dilators, the addition of Kampo medicines may reduce these side effects after reducing the dose of coronary dilators [34,36]. In general, Kampo medicines should be taken three times a day just before meals, but as reported for Keishibukurhyogan plus shigyakusan [37], starting with a small dose may also be effective and may be an option if three doses are not feasible.

Moreover, side effects should be also noted. Given the presence of licorice, side effects such as myopathy and pseudo-aldosteronism are likely to occur, and other serious side effects have also been reported. The safety of Mokuboito is not proven in patients with acute decompensated heart failure, accompanied by a low left ventricular ejection fraction and hypotension [27]. Besides, adverse events associated with Kampo formulas as immune allergic reactions, including interstitial pneumonia, limit their usage in patients with a previous history of serious allergic reactions [49]. As Saibokuto contains Shosaikoto, it is necessary to consider reducing the dose or switching to Hangekobokuto [34].

**Figure 1:**
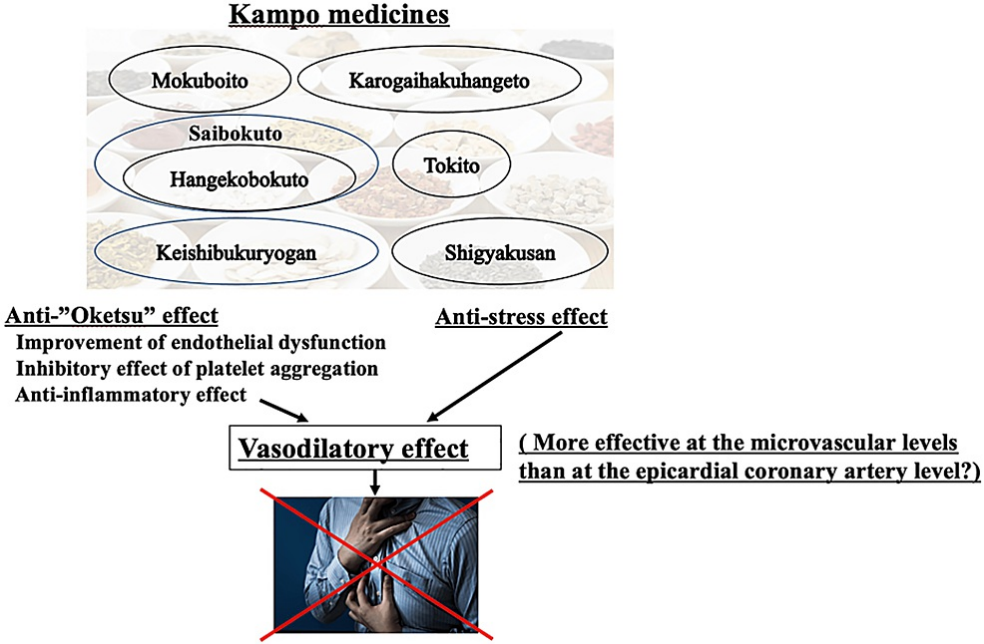
Relationship between Kampo medicines and relief in chest symptoms in patients with ischemia with non-obstructive coronary artery disease. Original photos were purchased from PIXTA Inc.

Essentially, it is not clear which of the herbal medicines is the best one to start with, but we need to test some of them for efficacy if possible. Although the effects are relatively rapid, it may take up to three months when starting with a small dose [37], and that may be the amount of time needed to confirm the effects. Personally, I think Keishibukuryogan plus Shigyakusan is currently the last resort. The problem is still that the case reports are mainly case reports showing efficacy, and only those that were effective would have been reported. It is unclear how effective Kampo is in the overall population of INOCA patients with persistent symptoms. In addition, the possibility that there are racial differences in efficacy cannot be ruled out, since the reports are from Japan only. Because of these limitations, multicenter and international registries and clinical studies may be needed. We would also like to see prospective clinical studies of Kampo medicines in patients with INOCA having refractory chest symptoms, just as clinical studies of Kampo medicines in heart failure have been initiated [28]. Among patients with VSA, given that patients who have CMD have a poor prognosis [1], medically refractory chest symptoms may occur in the presence of CMD, even in patients with INOCA. Thus, the presence of CMD, which can be assessed with MVFT, in such multicenter registries or prospective clinical trials should be clarified to better define which patients with INOCA will benefit from Kampo medicines (Figure *1*).

## Conclusions

Kampo medicines have the potential to improve thoracic symptoms in patients with INOCA, especially in those with microvascular abnormalities such as MVS and/or CMD. While Kampo medicines have the potential to improve chest symptoms in these patients with INOCA, its efficacy is limited to a few Japanese patients. It may be worthwhile to try Kampo medicines in patients with persistent chest symptoms despite current standard medical therapy in addition to lifestyle modification. In the future, it appears worthwhile to conduct a multicenter registry or prospective clinical trial after clarifying the presence of CMD to determine in which patients with INOCA Kampo medicines are effective.
